# NovoBoard: A Comprehensive Framework for Evaluating the False Discovery Rate and Accuracy of *De Novo* Peptide Sequencing

**DOI:** 10.1016/j.mcpro.2024.100849

**Published:** 2024-09-24

**Authors:** Ngoc Hieu Tran, Rui Qiao, Zeping Mao, Shengying Pan, Qing Zhang, Wenting Li, Lei Xin, Ming Li, Baozhen Shan

**Affiliations:** 1 Bioinformatics Solutions Inc, Waterloo, Ontario, Canada; 2 David R. Cheriton School of Computer Science, University of Waterloo, Ontario, Canada

**Keywords:** *de novo* peptide sequencing, mass spectrometry, false discovery rate, deep learning, neural network

## Abstract

*De novo* peptide sequencing is one of the most fundamental research areas in mass spectrometry–based proteomics. Many methods have often been evaluated using a couple of simple metrics that do not fully reflect their overall performance. Moreover, there has not been an established method to estimate the false discovery rate (FDR) of *de novo* peptide-spectrum matches. Here we propose NovoBoard, a comprehensive framework to evaluate the performance of *de novo* peptide-sequencing methods. The framework consists of diverse benchmark datasets (including tryptic, nontryptic, immunopeptidomics, and different species) and a standard set of accuracy metrics to evaluate the fragment ions, amino acids, and peptides of the *de novo* results. More importantly, a new approach is designed to evaluate *de novo* peptide-sequencing methods on target-decoy spectra and to estimate and validate their FDRs. Our FDR estimation provides valuable information to assess the reliability of new peptides identified by *de novo* sequencing tools, especially when no ground-truth information is available to evaluate their accuracy. The FDR estimation can also be used to evaluate the capability of *de novo* peptide sequencing tools to distinguish between *de novo* peptide-spectrum matches and random matches. Our results thoroughly reveal the strengths and weaknesses of different *de novo* peptide-sequencing methods and how their performances depend on specific applications and the types of data.

## Highlights

• We proposed a new method to estimate the FDR of *de novo* peptide sequencing.
• A framework consisting of benchmark datasets and accuracy metrics were presented.
• The results revealed the strengths and weaknesses of *de novo* sequencing tools.

## Graphical Abstract

**Figure:**
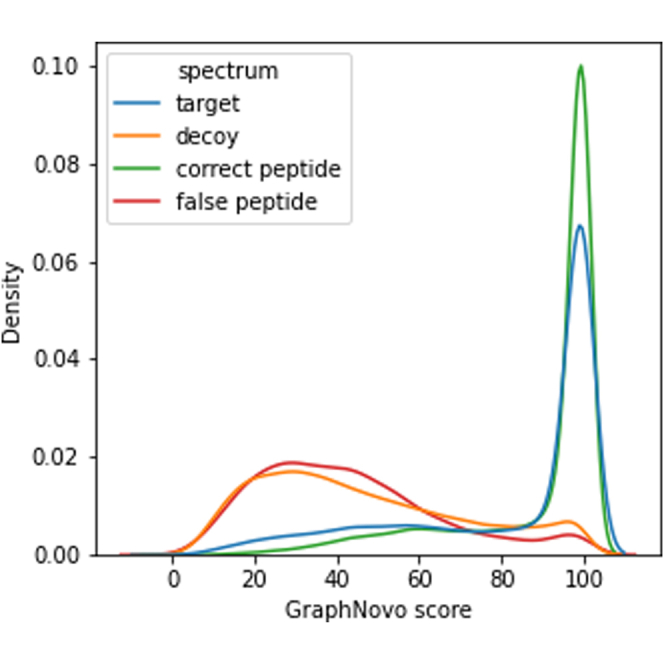

## In Brief

*De novo* peptide sequencing methods have often been evaluated using a couple of simple metrics that do not fully reflect their overall performance. Moreover, there has not been an established method to estimate their false discovery rate (FDR). We proposed NovoBoard, a comprehensive framework that includes diverse benchmark datasets, accuracy metrics, and a new approach to estimate and validate the FDR for *de novo* peptide sequencing. Our FDR estimation provides valuable information to assess the significance of *de novo* peptides.

*De novo* peptide sequencing is essential for the identification of novel peptides or proteins that do not exist in any databases, such as mutated neoantigens or antibody variable regions (1). This has been an intensive research area in mass spectrometry (MS)-based proteomics, with dozens of algorithms proposed over the last 3 decades (2, 3, 4, 5, 6, 7, 8, 9, 10, 11, 12, 13). In addition to popular, long-standing tools such as PEAKS (6), Novor (7), pNovo (14), there is a recent rise of deep learning-based *de novo* sequencing models, with over 20 new methods proposed in the last 5 years. Some prominent names include DeepNovo (5, 15), SMSNet (12), PointNovo (8), Casanovo (9, 10), PepNet (13), GraphNovo (11), etc. A comprehensive overview of deep learning methods for *de novo* peptide sequencing can be found in a recent review paper by Bittremieux *et al*. (16).

However, a fundamental problem, but often overlooked or simplified, is how to evaluate and benchmark the performance of *de novo* peptide sequencing. For instance, despite having a long history as the database search approach, *de novo* peptide sequencing still does not have a standard method to estimate the false discovery rate (FDR), as opposed to its counterpart. In the database search approach, peptides are acquired by *in silico* digesting the protein sequences from the database of interest, and we only need to assess the FDR of their peptide-spectrum matches (PSMs). This is often done by first creating a decoy protein database, either by randomly shuffling or reversing the target protein database. A spectrum is then compared to *in silico*–digested peptides from both target and decoy databases to identify its best matching peptide. All PSMs in the dataset are then sorted by their identification scores and the FDR is estimated as the proportion of decoy PSMs with scores above a cutoff (17). However, for *de novo* peptide sequencing, it is not clear how to generate appropriate decoys. Furthermore, a *de novo* peptide may still contain sequence errors even when its PSM has a very high score. Thus, FDR estimation is challenging for *de novo* peptide sequencing.

In the absence of FDR estimation, most existing studies evaluate *de novo* peptides by comparing them to ground-truth peptides and calculating their peptide and amino acid accuracies. Evaluation datasets often consist of PSMs obtained from the database search results of common organisms such as human, mouse, yeast, etc. Database peptides identified at strict FDR levels of 1%, or 0.1%, are considered as ground-truth to evaluate the *de novo* peptides identified on the same spectra. However, such an evaluation approach suffers from several major limitations. First, the definitions and calculations of those accuracy metrics might slightly differ from one study to another, thus making it difficult to obtain consistent evaluation results. Second, the evaluation spectra identified at those strict FDR levels are more likely to have very good fragmentations and high signal-to-noise ratios. They often account for less than 20 to 30% and are not a good representation of the whole dataset, especially for spectra of medium to low quality where unknown peptides may come from. In addition, the evaluation peptides are also highly biased because they come from well-known databases that have been analyzed numerous times. Thus, the peptides and spectra obtained from the database search may be very different from the unknown peptides and spectra that a *de novo* sequencing algorithm is supposed to find. More importantly, this evaluation approach cannot be applied when there is no database search ground truth.

Another challenge arises from recent deep learning and large-language models (LLMs) developed over the last few years. Those models are purposely designed to learn much more patterns of fragment ions and amino acid sequences to predict more accurate peptides. However, it is also their impressive learning capability that may lead to overfitting and memorizing, rather than generalizing and discovering new sequences. Some efforts have been put into controlling the overfitting issue, such as splitting the data into training and testing sets with nonoverlapping peptide sequences or using two different species for training and testing. However, due to the nature of high similarity of genomic and protein sequences, two different proteins or peptides may still share sub-sequences. Thus, information from the training may be leaked to the evaluation. It is not trivial how to address this problem, especially when the models and the data are getting larger and larger.

Thus, a comprehensive framework with carefully designed metrics and FDR estimation to evaluate the performance of *de novo* peptide sequencing is highly desired, now more than ever.

## Experimental Procedures

### Experimental Design and Statistical Rationale

We carefully collected different types of datasets that represent a wide range of applications of *de novo* peptide sequencing. The Association of Biomolecular Resource Facilities (ABRF) dataset was obtained from the 2017 ABRF Proteomics Research Group study. This is a standard human tryptic dataset where most *de novo* sequencing methods should achieve very good performance. The nontryptic dataset from Wang *et al*. (18) (PXD010154) includes six enzymes: Arg-C, Asp-N, Chymotrypsin, Glu-C, Lys-C, and Lys-N. Since many *de novo* sequencing models have been trained mainly on tryptic data, it is important to assess how they would perform on nontryptic data. The *A. thaliana* dataset from Muntel *et al*. (19) (PXD013658) was included to evaluate *de novo* sequencing methods on a species that is less related to human and other common model organisms. To the best of our knowledge, *A. thaliana* data has not been used to train any *de novo* sequencing models. We also performed evaluation on an HLA class 1 dataset from Wilhelm *et al*. (20) (PXD021013). MS-based immunopeptidomics is a major application of *de novo* peptide sequencing since this technique is essential to identify mutated neoantigens. In addition, due to their unspecific digestion and other unique characteristics such as length and binding motifs, HLA peptides are very different from those peptides derived from trypsin and other enzymes’ digestion.

In addition to previously published datasets, we also generated new datasets for evaluation. Three tryptic datasets from three species *Escherichia coli (E. coli)*, *Saccharomyces cerevisiae* (yeast), and *Homo sapiens* (human) were generated under the same experimental conditions and instrument. They would be used to investigate the issue of “memorizing peptide sequences” of *de novo* sequencing models, that is, whether those models were more biased towards human peptides than to other species. Finally, we simulated a dataset of mutated peptides and their *in silico* spectra to evaluate *de novo* sequencing methods on their capability to identify mutations. The new datasets generated in our study can be obtained from the ProteomeXchange Consortium *via* the PRIDE (21) partner repository with the dataset identifier PXD055277.

### Sample Preparation and LC-MS/MS Analysis for Human, Yeast, and *E. coli* Samples

Human and yeast protein digestion samples were purchased from Promega Corporation (V6951 and V7461). *E. coli* protein digestion standard was purchased from Waters Corporation (186003196). All reagents used were LC-MS grade and purchased from Sigma-Aldrich Corporation. All protein digestion samples were dissolved in 0.1 formic acid to 1 μg/μl for LC-MS/MS analysis.

Two microliters of each sample was injected into the Thermo Orbitrap Exploris 240 (Thermo Fisher Scientific) by nanoflow liquid chromatography using a Ultimate 3000 chromatography system (Thermo Fisher Scientific). Liquid chromatography was performed using a constant flow of 0.25 μl/min and a 15 cm reversed-phased column with a 75 μm inner diameter filled with Reprosil C18 (PepSEP). Mobile phase A was 0.1% formic acid and mobile phase B was 99.9% acetonitrile, 0.1% formic acid. The separation was carried out over 60 min as follows; linearly 4% B to 35% B over 53 min with an increase to 95% B over 0.1 min and held constant for 2.9 min to clean the column. Then B percentage was set back to 4% in the final 4 min.

MS data were acquired on Thermo Orbitrap Exploris 240 in data-dependent mode with a cycle time of 3 s. MS1 scan data were obtained at 60,000 resolution (at 400 m/z) with a mass range of 400–1600 m/z. The automatic gain control was set to standard, with an auto maximum ion injection time. The radio frequency lens was set to 70%. Isolation for MS2 scans was performed in the quadrupole, with an isolation window of 0.7. MS2 scan data were acquired at a resolution of 15,000 m/z, with a standard automatic gain control target and an auto ion injection time. The scan range of MS2 was also set to auto. Higher energy collisional dissociation (HCD; fixed normalized collision energy: 30%) was used for generating MS2 spectra, with the number of microscans set to 1. The dynamic exclusion was set as 8 s.

### Raw Data Processing and Protein Database Search

The raw data of all datasets were converted to MGF files using the Proteome Discoverer software (version 3.1). Protein database search was performed using Proteome Discoverer (version 3.1) and PEAKS Studio (version 12). The following parameters were used for protein database search: precursor mass tolerance 10 ppm, fragment ion mass tolerance 0.02 Da, cysteine(Carbamidomethylation) as fixed modification, and methionine(Oxidation) as variable modification. For the HLA dataset, enzyme specificity was set to unspecific. The protein databases were obtained from the UniProtKB/Swiss-Prot database, including human (version 20240224, 20435 entries), *A. thaliana* (version 20240224, 16,389 entries), *E. coli* (version 20240807, 4530 entries), and yeast (version 20240807, 6727 entries). The peptide FDR was set to 1%.

### Models and Parameters for *De Novo* Peptide Sequencing Tools

In this study, we considered five *de novo* sequencing tools: PEAKS (6) (version 12), PointNovo (8), Casanovo (9, 10) (version 4.1.0), GraphNovo (11), and Novor (7). PointNovo was run in PEAKS Studio (version 11) and GraphNovo was run in PEAKS Studio (version 12). Novor was run using their web server (https://novor.cloud/). It should be emphasized that the evaluation framework proposed here can be easily applied to any other tools. In fact, we expect that when more tools are included in the evaluation, new benchmark datasets and metrics will emerge, as different tools may have different strengths and weaknesses. There are over 20 *de novo* sequencing methods proposed in the last 5 years, and a comprehensive overview of them can be found in a recent review paper by Bittremieux *et al*. (16).

PEAKS is one of the most common tools for *de novo* peptide sequencing and has been actively developed and maintained over the last 20 years. In addition, PEAKS is based on a traditional dynamic programming algorithm as opposed to recent deep learning-based methods, thus it could serve as a robust baseline for our evaluation study. Similar to PEAKS, Novor is also a popular nondeep learning tool for *de novo* peptide sequencing and is based on a decision tree algorithm.

PointNovo and its predecessor DeepNovo (5, 15) were among the first deep learning models developed for *de novo* peptide sequencing. They have proved that deep learning models are capable of learning considerably more features from the MS data and offer substantial improvements over traditional methods, both in terms of accuracy and methodology. Deep learning models also have the advantages over traditional methods since the data is growing exponentially.

Casanovo model is based on the Transformer (22) neural network, the popular architecture that powers most of today’s large-language models. The impressive learning capability of Transformer has been demonstrated on large-scale models and data, such as GPT-4 (23), Llama (24), etc, especially on text data. Thus, one could expect that such a model may also show great performance on protein sequence data, which can be viewed as a special kind of language.

GraphNovo is a recent *de novo* peptide sequencing model that is based on a graph neural network. It is designed to combine the advantages of both deep learning and graph theory, a popular approach to model the relationships between fragment ions in an MS/MS spectrum (4). By carefully modeling the features of the fragment ions (nodes) and their relationships (edges) in a spectrum graph, GraphNovo can address the missing-fragmentation problem in *de novo* peptide sequencing and subsequently improve the sequencing accuracy over previous methods.

GraphNovo model was trained on the MassIVE-KB database (25) as mentioned in Mao *et al*. (11) and was applied to all datasets in the study. PointNovo had a tryptic model, which was trained on MassIVE-KB, and an HLA model, which was trained on a large peptidome dataset from Sarkizova *et al*. (26). The tryptic model was applied to the tryptic datasets and the HLA model was applied to the nontryptic and the HLA datasets. Similarly, Casanovo also had a tryptic model trained on MassIVE-KB and a nontryptic model (9). The tryptic model was applied to the tryptic datasets and the nontryptic model was applied to the nontryptic and the HLA datasets. PEAKS and Novor used their own internal algorithms; there was no model training or selection involved.

All *de novo* sequencing tools were run on the MGF files of the evaluation datasets. The *de novo* sequencing parameters were set similar to those used in the database search above. No score filter was applied to the output *de novo* results. PEAKS, PointNovo, Casanovo, and GraphNovo were run on a machine equipped with an Intel CPU (i9-13900K), an NVIDIA GPU (GeForce RTX 4090), and 128 GB memory. PEAKS, PointNovo, and GraphNovo were run in PEAKS Studio in Windows environment, while Casanovo was run in Linux environment. Novor was run using their web server (https://novor.cloud/).

### Calculation of Fragment Ion, Amino Acid, and Peptide Accuracy Metrics for *De Novo* Peptides

Given a tandem mass spectrum and its ground-truth peptide, a *de novo* peptide predicted from that spectrum was compared to the ground-truth peptide as follows.

The masses of the theoretical ions of both *de novo* and ground-truth peptides were calculated from their amino acid sequences. Twelve types of theoretical ions were considered: b/y, b/y-H2O, b/y-NH3, with charge 1 and charge 2 for each of them. The theoretical ions were then compared against all of the peaks in the spectrum. A matched ion was confirmed if the mass difference between a theoretical ion and a peak was less than or equal to 0.02 Da. We then calculated the number of matched ions for the ground-truth peptide, for the *de novo* peptide, and the number of shared matched ions between them. The calculation was repeated across all PSMs of the dataset to obtain the total numbers of matched ions. The fragment ion accuracy was then calculated as the proportion of the total number of shared matched ions over the total number of matched ions of the ground-truth peptides.

The amino acid accuracy was calculated using the approach proposed in Ma (7) and implemented in Tran *et al*. (5). In particular, when comparing a *de novo* peptide to a ground-truth peptide, a *de novo* amino acid was considered matched with a ground-truth amino acid if their masses were different by less than 0.1 Da and if the prefix masses before them were different by less than 0.5 Da. Such an approximate match was used instead of an exact match because the resolution of mass spectrometers might not be sufficient to distinguish isobaric amino acid combinations with nearly identical masses (*e.g.* I *versus* L, N(Deamidation) *versus* D, K *versus* Q, Q(Deamidation) *versus* E, M(Oxidation) *versus* F, etc.). The comparison was performed by simultaneously iterating over the *de novo* peptide sequence and the ground-truth peptide sequence from N-terminal to C-terminal. If all of the *de novo* amino acids were correctly matched, the *de novo* peptide was considered matched with the ground-truth peptide. The numbers of matched *de novo* amino acids and peptides were summed up over all PSMs in the dataset. They were then divided by the total numbers of ground-truth amino acids and peptides to obtain the amino acid and peptide accuracies, respectively.

The Python scripts for calculating the fragment ion, amino acid, and peptide accuracy metrics are available in the Data Availability section.

### Detailed Procedures for FDR Estimation and Validation for *De Novo* Peptide Sequencing

#### Generating Decoy Spectra

Given an input dataset of tandem mass spectra, here named “target spectra”, a “decoy” spectrum was generated for every target spectrum as follows. Each peak in a spectrum was defined as a pair of (m/z, intensity). The generating process included (1) removing a number of peaks from the target spectrum, named “target peaks” and (2) adding back the same number of “noise peaks” to create the decoy spectrum. Two following strategies were applied to select the target peaks for removal.

In the first strategy, named random peak removal, we first went through all target spectra to collect all of their peaks to create a peak list, named *P*. Then for each target spectrum, we randomly removed an *X*% of its peaks, where *X*% was an input parameter. Subsequently, the same number of noise peaks were randomly sampled from the peak list *P* and added back to create the decoy spectrum. As a result, each decoy spectrum had the same number of peaks as its target spectrum, and furthermore, the target peaks and the noise peaks came from the same distribution of the peak list *P* (so each noise peak was actually a real peak, *i.e.* originating from a real peptide fragment ion). Different values of *X*%, from 10 to 90%, were used to generate decoy spectra to investigate its impact on the FDR estimation.

The second strategy was peak removal by intensity and peptide mass. First, the number of peaks to be removed from a target spectrum was determined as follows. The peptide neural mass *M* (Da) was calculated from the precursor mass and the charge. Assuming that the average mass of standard amino acids was about 123 (Da), we estimated the peptide length as *L = M/123*, the expected number of theoretical b/y ions as *N*_*ions*_*=(L-1)∗2*, and the number of removed peaks as *N*_*peaks*_ *= N*_*ions*_*∗X%*, where *X%* varied from 10 to 90%. The top *N*_*peaks*_ peaks with the highest intensities from the target spectrum were removed and replaced by the same number of noise peaks. The noise peaks were randomly sampled from all the peaks that had been removed from all target spectra.

The MGF files of decoy spectra are available in the supplementary materials of this study (see Data Availability section).

#### Target-Decoy Competition and FDR Estimation

Given an input dataset of tandem mass spectra, a decoy spectrum was generated for every target spectrum as described above. *De novo* peptide sequencing was performed on all target and decoy spectra. As a result, for each MS/MS scan id, there were two *de novo* PSMs, one from the target spectrum and the other from the decoy spectrum of that scan id. Two target-decoy competition approaches were then considered. In the first approach, for each MS/MS scan id, only the higher scoring PSM between the target and decoy *de novo* PSMs of that scan was kept, the other was discarded. In the second approach, both target and decoy *de novo* PSMs of each MS/MS scan id were kept (so the total number of *de novo* PSMs would be twice the number of input MS/MS scans). The rationale behind the second approach was that we believed it would provide a larger competition space by allowing a decoy spectrum to compete with other target spectra rather than just its own target counterpart, and thus it might lead to more stringent FDR estimation. Finally, the *de novo* PSMs were sorted according to their scores, and for any given score cutoff, the FDR was estimated as the proportion of decoy PSMs with scores above that cutoff.

#### Definition of True FDR for Validating the Estimated FDR

To validate the estimated FDR, we need to define what the true FDR is. In this study, we defined the true FDR as the “false peptide rate,” which was calculated on the spectra with known ground-truth peptides. We proposed the following definition of “correct” *de novo* peptide based on the fragment ion information:

• The spectrum was compared to the ground-truth peptide to find matched fragment ions, named set 1
• The spectrum was compared to the *de novo* peptide to find matched fragment ions, named set 2
• If set 2 covered more than *Y%* of set 1, then the *de novo* peptide was considered “correct.” Otherwise, it was considered a “false” identification.

Note that this definition of “correct” *de novo* peptide was only used to calculate the true FDR based on the fragment ion information. It was not related to the peptide accuracy metric described earlier. The rationale behind this definition shall be discussed in the Results section below. In this study, *Y%* was set to 90% by default. This parameter allows users to relax the criteria of the true FDR, according to their expectation of *de novo* sequencing results, which may depend on the type of data, instruments, fragmentation techniques, etc.

The Python scripts for generating decoy spectra, FDR estimation, and validation are available in the Data Availability section.

### Simulating Dataset of Mutated Peptides and Their *In Silico* Spectra

Ten thousand peptides were randomly selected from the MassIVE-KB database (25). Subsequently, amino acid substitutions were introduced to each peptide, where the mutation locations and the substituted amino acids were randomly selected. The number of mutations per peptide varied from 1 to 10, and ten different datasets were generated accordingly (*i.e.* dataset 1 contained peptides with 1 mutation, dataset 2 contained peptides with 2 mutations, etc.). Finally, the spectra of those mutated peptides were predicted using Prosit (20) with their 2020 HCD model. The output files from Prosit were converted to MGF format for further analysis. The MGF files of mutated peptides are available in the supplementary materials of this study (see Data Availability section).

## Results

In this study, we proposed NovoBoard, a new framework that included benchmark datasets, accuracy metrics, and FDR estimation to evaluate the performance of *de novo* peptide sequencing methods. The comprehensive details of the framework have been described in the Experimental Procedures section. In this section, we first highlight two of our new contributions, namely fragment ion accuracy and FDR estimation, and then present the evaluation results of our framework.

### Fragment Ion Accuracy for *De Novo* Peptide Sequencing

Peptide and amino acid accuracies are the two standard evaluation metrics that have been used in most previous studies. Here we proposed a new metric named fragment ion accuracy and its motivation is as follows. Deep learning models are capable of learning many features, including some beyond the information provided by the mass spectra, such as the frequency or the sequential patterns of amino acids in the training data. While those extra features are helpful, and sometimes very powerful, we argue that a *de novo* peptide predicted from a tandem mass spectrum must have sufficient supporting evidence from that spectrum, that is, the supporting fragment ions. Thus, when comparing a *de novo* peptide to a ground-truth on a tandem mass spectrum, we also counted how many peaks in the spectrum matched the theoretical ions of both *de novo* and ground-truth peptides. The fragment ion accuracy was then calculated as the percentage of theoretical ions of the ground-truth that were observed in both the *de novo* peptide and the spectrum (see the Experimental Procedures section for more details). We believe this new metric provides a fair evaluation of *de novo* peptide sequencing tools to identify relevant fragment ions from the tandem mass spectra.

### Estimating the FDR for *De Novo* Peptide Sequencing

The three accuracy metrics above, however, still rely on a ground-truth peptide for every tandem mass spectrum. As mentioned in the introduction, the ground-truth peptides and PSMs are often obtained from the database search results, which may be biased towards high-quality spectra and well-known protein databases. More importantly, those metrics cannot measure how strong a *de novo* peptide-spectrum match is compared to a random match. Thus, an FDR estimation is desired to evaluate the *de novo* results.

In the database search approach, decoy peptides can be generated by randomly shuffling or reversing the target protein database. However, since most *de novo* peptide-sequencing methods are designed to find the optimum amino acid sequence for a given tandem mass spectrum, a random decoy peptide is not likely to yield a better match to that spectrum. Thus, we decided to use decoy spectra rather than decoy peptides to estimate the FDR for *de novo* peptide sequencing.

**Fig. 1:**
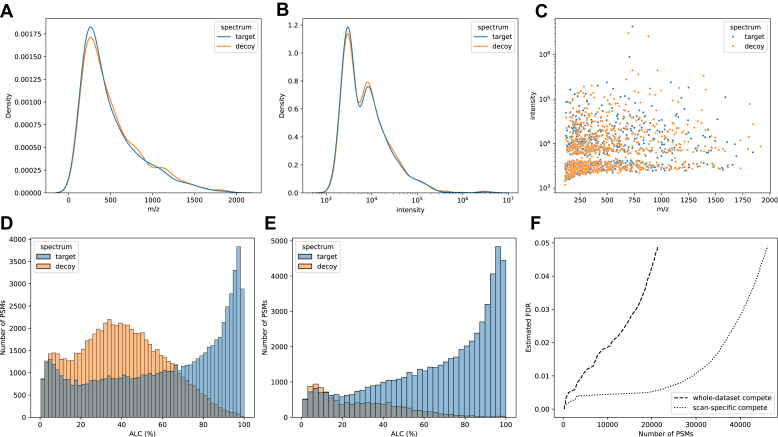
**Generating decoy spectra and performing target-decoy competition for FDR estimation.***A*–*C*, m/z and intensity distributions of the peaks in the target and decoy spectra of the ABRF dataset. For each scan id, one decoy spectrum was generated from the target spectrum by removing 50% of the target peaks and then adding the same number of noise peaks. Noise peaks were drawn from the same distribution of the target peaks. *D*, distributions of PEAKS *De novo* scores, ALC (%), on 70K target and 70K decoy spectra of the ABRF dataset. *E*, for each scan id, the higher scoring PSM between the target and decoy spectra was selected. As a result, there were 70K *de novo* PSMs remained after the selection, one for each scan id. *F*, FDR was estimated as the proportion of decoy PSMs with scores above a given threshold from the distribution in (*D*) (whole-dataset compete) or the distribution in (*E*) (scan-specific compete).

Given an input dataset of tandem mass spectra, we generated a decoy spectrum for every target spectrum following a process that included (1) removing a number of peaks from the target spectrum and (2) adding back the same number of noise peaks to create the decoy spectrum (here a peak is defined as a pair of m/z and intensity values). The process ensured that every decoy spectrum had the same number of peaks as its target spectrum and the noise peaks were randomly drawn from the same distribution as the target peaks. For instance, Figure 1, *A*–*C* show the m/z and intensity distributions of the peaks in the target and decoy spectra of the ABRF dataset, which were nearly identical. Another key question was to determine how many peaks that we should remove and how they should be selected. We experimented with different percentages of removed peaks, from 10 to 90%, and we also considered two strategies to select the target peaks to remove. The first strategy was random peak removal, that is, peaks were randomly removed regardless of their m/z or intensity values. In the second strategy, the number of peaks to remove was calculated to mimic the expected number of theoretical b/y ions derived from the peptide mass and the peaks were then selected by their intensities. More details of the decoy spectra generating process can be found in the Experimental Procedures section.

To investigate whether the decoy spectra that we generated were too “easy” and too “hard” to distinguish from the target ones, we performed *de novo* peptide sequencing on the decoy spectra and calculated the peptide accuracy. Supplemental Fig. S1*A* shows the *de novo* peptide accuracy on the decoy spectra generated by random peak removal. The *de novo* peptide accuracy dropped when the percentage of removed peaks increased, and when 60% or more of the target peaks were removed, the *de novo* peptide accuracy dropped to near zero. Supplemental Fig. S1*B* demonstrates the effect of different strategies to remove peaks. The strategy of removing peaks based on the intensities and peptide mass generated “harder” decoy spectra, that is, with higher *de novo* peptide accuracy than the strategy of random peak removal. Even when we increased the removal percentage to 90%, the *de novo* peptide accuracy was still near 10%, indicating that decoy spectra still contained sufficient information to predict the peptides. For further FDR estimation, we shall consider all of those strategies and percentages to investigate their impacts on the FDR estimation.

Once the decoy spectra had been generated, *de novo* peptide sequencing was performed on all target and decoy spectra, and their *de novo* score distributions were compared for FDR estimation (Fig. 1*D*). However, since there were two *de novo* PSMs for each MS/MS scan id, one from the target spectrum and the other from the decoy spectrum, one might also want to choose the higher scoring PSM to keep and discard the other (Fig. 1*E*). In this study, we preferred to estimate the FDR from the distributions in Figure 1*D* than from those in Figure 1*E*, because the formers provided a larger competition space by allowing a decoy spectrum to compete with other target spectra rather than just its own target counterpart. Finally, the *de novo* PSMs were sorted according to their scores, and for any given score cutoff, the FDR was estimated as the proportion of decoy PSMs with scores above that cutoff. Figure 1*F* indeed confirmed that the target-decoy competition from Figure 1*D* led to more stringent FDR estimation than the one from Figure 1*E*.

### Validating the Estimated FDR for De Novo Peptide Sequencing

To validate the estimated FDR, we first need to define what the true FDR is. For instance, the true FDR could be defined as the “false peptide rate,” which was calculated on the spectra with known ground-truth peptides, for example, those identified by a database search engine. Then a *de novo* peptide could be considered correct if it matched the ground-truth peptide, otherwise it was considered as a false identification. However, since a database search engine only needs a handful of matched fragment ions to identify a peptide, some identified peptides actually do not have a full series of supporting b/y ions presented in their spectra. In such cases, it is unfair to ask a *de novo* sequencing tool to predict the exact amino acid sequence if the required fragment ion information is simply not available in the spectrum. In addition, our process of generating decoy spectra and subsequent FDR estimation was mainly based on the fragment ion information. Thus, we proposed to relax the definition of “correct” *de novo* peptide as follows: a *de novo* peptide was considered “correct” if it covered more than a predefined percentage, *Y%,* of the fragment ions of the ground-truth peptide in the spectrum. In this study, *Y%* was set to 90% by default. This parameter allows users to relax the criteria of the true FDR, according to their expectation of *de novo* sequencing results, which may depend on the type of data, instruments, fragmentation techniques, etc. More details of the true FDR calculation can be found in the Experimental Procedures section.

**Fig. 2:**
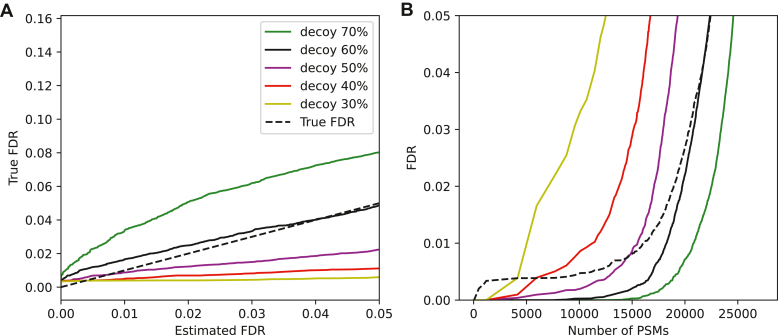
**FDR estimation and validation of GraphNovo using the decoy spectra generated from the ABRF dataset.** In this example, the decoy spectra were generated by random peak removal. Different colors indicate different percentages of removed peaks, for instance, decoy 70% (*green*) means that we removed 70% of the original peaks in a target spectrum and replaced them with noise peaks to generate the corresponding decoy spectrum. *A*, the estimated FDR *versus* the true FDR with respect to different GraphNovo score cutoffs. *B*, the number of *de novo* PSMs *versus* the estimated FDR and the true FDR with respect to different GraphNovo score cutoffs.

Figure 2 shows an example of the FDR estimation and validation from GraphNovo results on the ABRF dataset. Different colors correspond to different percentages of removed peaks, for instance, decoy-70% (green) means that we removed 70% of the target peaks in a target spectrum and replaced them with noise peaks to generate the corresponding decoy spectrum. In this example, peak removal was performed randomly. Figure 2*A* shows the estimated FDR *versus* the true FDR, which was calculated based on the fragment ion information as described above. In an ideal situation, the estimated FDR should be equal to the true FDR (black dashed line). In this example, the FDR estimated from decoy-60% spectra (black) was closest to the true FDR. The corresponding score distributions of the decoy-60% PSMs and the target PSMs with correct and false *de novo* peptides are shown in the Supplemental Fig. S2. The results from decoy-70% spectra (green) was an underestimation (the true FDR was higher than the estimated one), whereas the results from decoy-50% spectra (purple) was an overestimation. Figure 2*B* shows the number of *de novo* PSMs with respect to different FDR thresholds and different percentages of removed peaks in decoy spectra.

Having established the procedures for generating decoy spectra, FDR estimation, and validation, we then compared the FDRs of the *de novo* sequencing tools. The comparison was performed using two approaches of peak removal, that is, random and by intensity and peptide mass and using different percentages of removed peaks from 10 to 90%. Supplemental Fig. S3 shows the results obtained from the decoy spectra generated by random peak removal on the ABRF dataset. When comparing the estimated FDR to the true FDR, we observed that different *de novo* sequencing tools might behave differently on the generated decoy spectra. For instance, for decoy-50% spectra (purple), we got an accurate FDR estimation for PEAKS, an underestimation for PointNovo and Casanovo, and an overestimation for GraphNovo. We believe that these discrepancies were due to different algorithms and scoring functions implemented in the *de novo* sequencing tools. Nevertheless, one could select the most suitable percentage of removed peaks for each *de novo* sequencing tool, for example, 50% for PEAKS, 40% for PointNovo, 50% for Casanovo, and 60% for GraphNovo, to obtain an FDR estimation that was close to the true FDR. Novor was excluded from the FDR analysis because its cloud server did not accept our generated MGF files for decoy spectra.

We further performed a similar analysis on the decoy spectra generated by removing peaks based on their intensities and the peptide mass. Supplemental Fig. S4 again shows different behaviors of the *de novo* sequencing tools. We noticed that the differences between the estimated FDRs and the true FDRs were just less than 5%, smaller than those obtained from random peak removal. These results were consistent with our previous observation that the approach of removing peaks based on the intensities and peptide mass indeed generated “harder” decoy spectra (Supplemental Fig. S1*B*), thus leading to more stringent FDR estimation. However, this approach also led to the overestimation for PEAKS and GraphNovo, because their underlying algorithms (dynamic programming and spectrum graph) tended to perform well on those decoy spectra.

Overall, our analysis results show that it was possible to generate decoy spectra and obtain accurate FDR estimations that were close to the true FDRs. However, one needs to adjust the strategies and the parameters for decoy spectra generation depending on each *de novo* peptide-sequencing tool, because each tool has their own model and scoring method. We prefer the strategy of random peak removal as it is unbiased and one can simply select the most suitable percentage of removed peaks for each *de novo* sequencing tool.

### Evaluation of De Novo Peptide Sequencing Tools on Tryptic Data

**Fig. 3:**
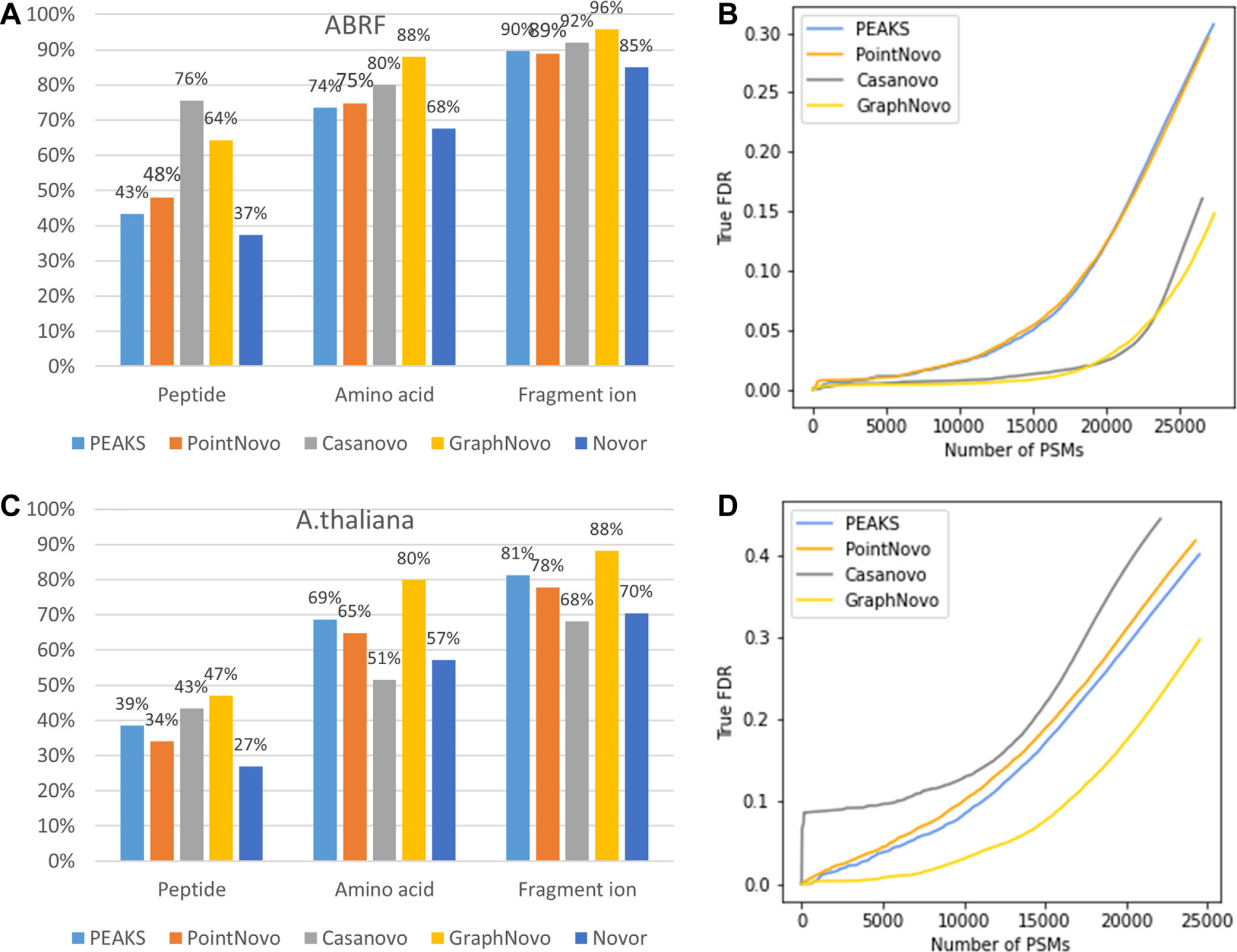
**Evaluation of the*****de novo*****sequencing results on the ABRFand the*****A. thaliana*****datasets.***AandB*, results on the ABRF dataset. *CandD*, results on the *A. thaliana* dataset.

We first evaluated the performance of the five *de novo* peptide-sequencing tools on the ABRF dataset. All three models PointNovo, Casanovo, and GraphNovo were trained on the MassIVE-KB human spectral library, which contains mostly tryptic peptides. Since the ABRF dataset is also human tryptic data, all five tools performed well on this dataset, with peptide, amino acid, and fragment ion accuracies at very high ranges of 37 to 76%, 68 to 88%, and 85 to 96%, respectively (Fig. 3*A*). The three deep-learning tools performed better than PEAKS and Novor as expected, especially on the peptide accuracy. On the amino acid and fragment ion metrics, GraphNovo outperformed the other tools, thanks to its intensive efforts in modeling the features of fragment ions, that is, the nodes in a spectrum graph (11). Casanovo achieved a very high peptide accuracy of 76%, while its amino acid and fragment ion accuracies were 4 to 8% lower than GraphNovo. This discrepancy suggests that Casanovo might perform exceptionally well on some peptides and predict the whole sequences correctly, but for some other peptides, it might not be able to make correct predictions.

We further compared the numbers of *de novo* PSMs reported by the *de novo* sequencing tools with respect to their FDRs. We first compared the numbers of *de novo* PSMs with respect to the true FDRs on the spectra with ground-truth peptides. Figure 3*B* shows that GraphNovo and Casanovo reported substantially more *de novo* PSMs than PEAKS and PointNovo at different FDR levels. Since the estimated FDRs varied across different sets of decoy spectra and different tools (Supplemental Fig. S3), we selected the percentages of removed peaks for each tool so that its estimated FDR was close to its true FDR, *i.e.* PEAKS 50%, PointNovo 40%, Casanovo 45%, and GraphNovo 60%. Supplemental Fig. S5 shows the numbers of *de novo* PSMs with respect to the estimated FDRs on all spectra of the ABRF dataset. GraphNovo and Casanovo again reported much more *de novo* PSMs than PEAKS and PointNovo. (FDR analysis was not available for Novor as its cloud server did not accept our generated MGF files for decoy spectra).

We next evaluated the performance of the *de novo* peptide sequencing tools on a tryptic dataset from *A. thaliana* (PXD013658, Muntel *et al*. (19)). *A. thaliana* is a species that is less related to human and to the best of our knowledge, it has not been used to train any *de novo* sequencing models. As expected, the accuracies of the *de novo* results dropped substantially when compared to the ABRF dataset, with peptide, amino acid, and fragment ion accuracies at 27 to 47%, 51 to 80%, and 68 to 88%, respectively (Fig. 3, *C* and *D*). While the performance drop was expected as the models were trained on human data, not *A. thaliana*, Casanovo suffered considerably. Its peptide accuracy decreased by 33% (76% to 43%) while its amino acid and fragment ion accuracies even fell behind all other tools. GraphNovo outperformed the other tools on all three accuracy metrics and also on the FDR evaluation. The results on this *A. thaliana* dataset represent a good validation test showing that deep learning models are biased towards the training data to different extents. It is also interesting to see that PEAKS performance was quite robust, as its peptide and amino acid accuracies only dropped around 4 to 5%, much less than the other three deep learning tools and Novor.

### Evaluation of *De Novo* Peptide Sequencing Tools on the Nontryptic and the HLA Datasets

Since tryptic peptides are over-represented in most public MS data repositories, including MassIVE-KB, *de novo* peptide sequencing tools may be biased towards those peptides. Thus, it is essential to assess how they would perform on nontryptic data. Here we performed evaluation on a nontryptic dataset from Wang *et al*. (18) that includes peptides digested from six enzymes: Arg-C, Asp-N, Chymotrypsin, Glu-C, Lys-C, and Lys-N. Casanovo has a nontryptic model that was fine-tuned on their MassIVE-KB model using the PSMs of peptides with diverse C-terminal amino acids obtained from MassIVE-KB and PROSPECT databases (9, 25) (https://zenodo.org/records/6602020). PointNovo has two models, one trained on MassIVE-KB and another trained on HLA data; we tested both and reported the results of the HLA model here since it showed better performance. For GraphNovo, we still used the same model trained on MassIVE-KB as there is no other option.

**Fig. 4:**
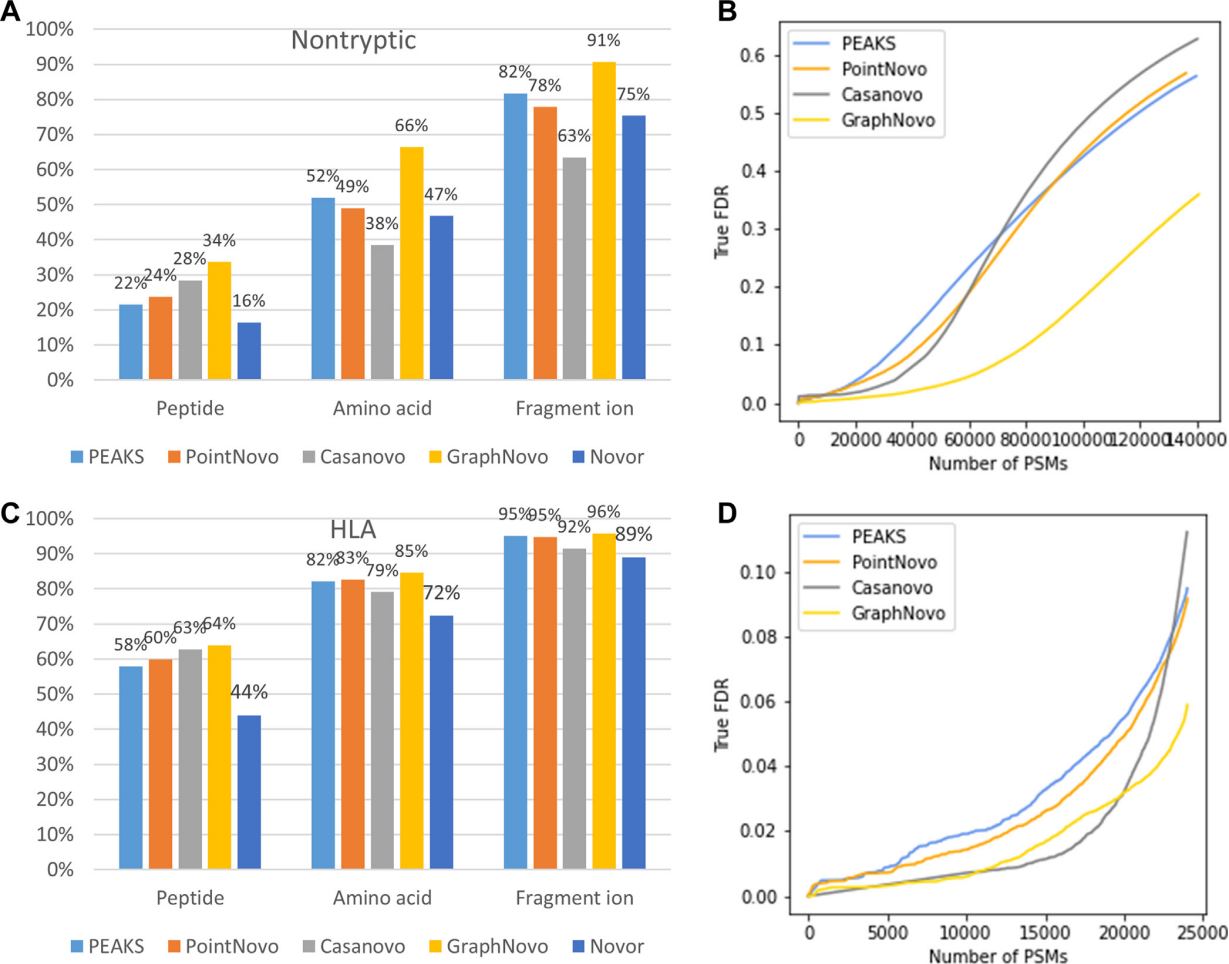
**Evaluation of the*****de novo*****sequencing results on the nontrypticand the HLA datasets.***AandB*, results on the nontryptic dataset. *CandD*, results on the HLA dataset.

The nontryptic evaluation results are shown in Figure 4, *A* and *B*. The overall performance of all tools are considerably lower than the previous results on tryptic data (Fig. 3). GraphNovo outperformed the other tools by a large margin; its peptide, amino acid, and fragment ion accuracies were 6%, 14%, and 9%, respectively, higher than the second-best tools. This is despite the fact that the GraphNovo model was trained on tryptic data, whereas PointNovo and Casanovo models had been fine-tuned on nontryptic data. The robust performance of GraphNovo over the other tools was also confirmed in the FDR evaluation (Fig. 4*B*). The outperformance of GraphNovo might seem somewhat unexpected as it was trained on tryptic data in contrast to the other models. We further checked the overlap between the nontryptic dataset and the MassIVE-KB database. About 28% of the nontryptic PSMs were found in the MassIVE-KB, this is because the dataset includes Arg-C and Lys-C, which may also produce tryptic peptides. We further excluded those PSMs from the nontryptic dataset and re-run the evaluation. As shown in the Supplemental Fig. S6, GraphNovo still performed better than the other tools. We believe that GraphNovo’s focus on fragment ions and spectrum graph helped it to generalize better to nontryptic peptides.

We further performed evaluation on an HLA class 1 dataset from Wilhelm *et al*. (20). HLA peptides are very different from tryptic peptides due to their unspecific digestion and other unique characteristics such as length and binding motifs. As shown in Figure 4*C*, except for Novor, the other four tools performed well on this dataset with very high peptide accuracy from 58 to 64%. This is probably because HLA class 1 peptides are short, with lengths from 8 to 12 amino acids, hence it is easier for the *de novo* peptide sequencing tools to predict the whole peptide sequences correctly. On the FDR evaluation, GraphNovo and Casanovo reported the most number of *de novo* PSMs, followed by PointNovo and PEAKS. To further investigate the impact of peptide lengths on the *de novo* sequencing, we re-evaluated the results on the ABRF dataset stratified by peptide lengths. As shown in the Supplemental Fig. S7, the accuracies decreased as the peptide length increased. The performance difference among the tools was also smaller on short peptides than on long peptides.

### Evaluation of *De Novo* Sequencing Tools on Mutated Peptides

In this section, we evaluated the *de novo* sequencing tools on a dataset of 10 thousand mutated peptides. The peptides were randomly selected from the MassIVE-KB database and 1 to 10 amino acid substitutions were randomly introduced to each peptide. Their spectra were then predicted using Prosit (20) with its 2020 HCD model. The deep learning models of PointNovo, Casanovo, and GraphNovo were all trained on the MassIVE-KB database. Thus, by starting with MassIVE-KB peptides and gradually increasing the number of mutations, we can investigate the impact of mutations on the performance of each tool.

**Fig. 5:**
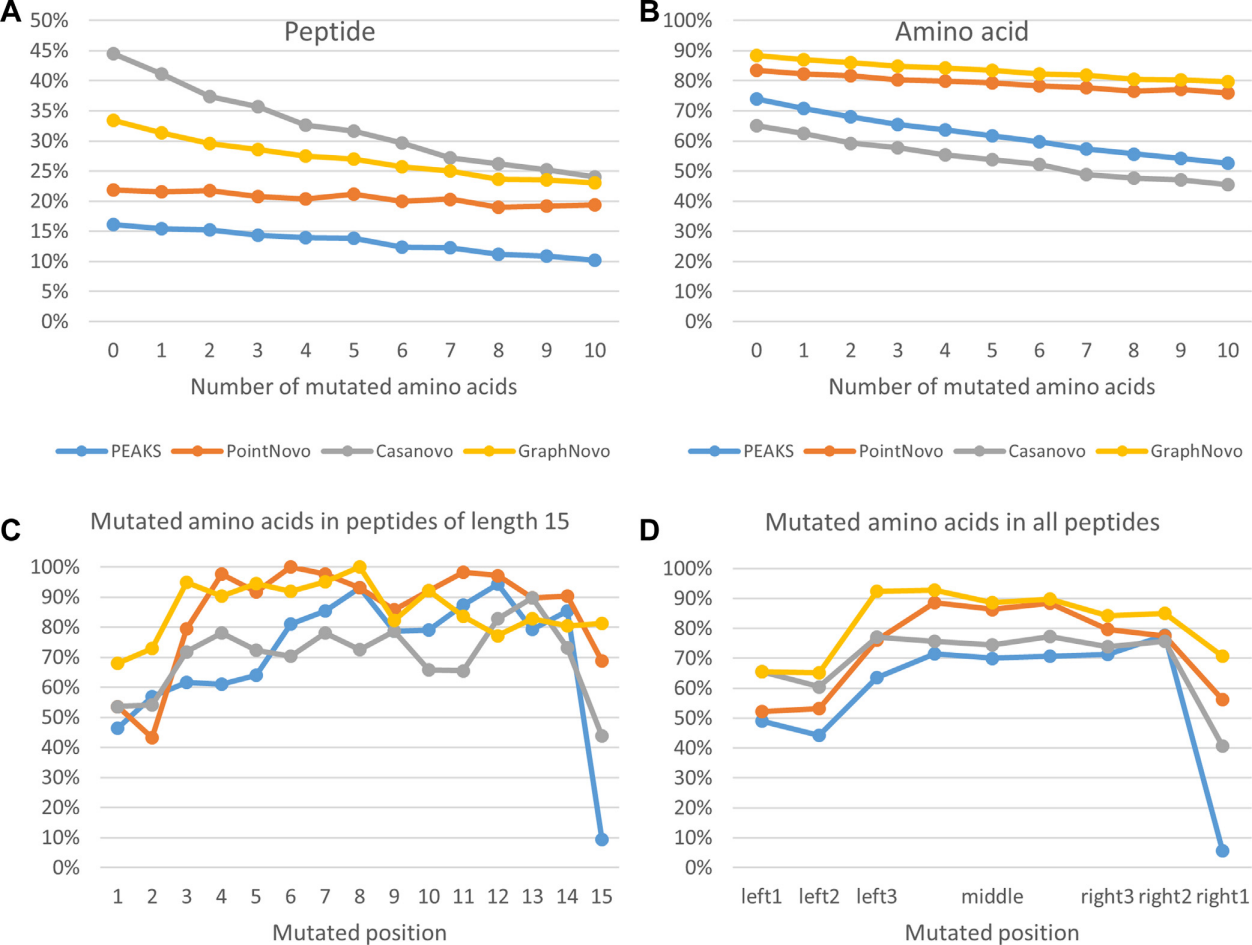
**Evaluation of the *de novo* results on the simulated dataset of mutated peptides.***A* and *B*, peptide and amino acid accuracies. *C* and *D*, mutated amino acid accuracy in peptides of length 15 and in all peptides.

Figure 5, *A* and *B* shows the peptide and amino acid accuracies of the *de novo* results with respect to the number of mutations. As expected, the overall performance decreased as the number of mutations increased from 0 to 10. On the peptide metric, Casanovo showed the steepest decrease of 21% (from 45% to 24%), followed by GraphNovo (from 33% to 23%) and PEAKS (from 16% to 10%), while PointNovo only showed minor decline (from 22% to 19%). On the amino acid metric, GraphNovo and PointNovo showed a modest decrease of about 8%, while Casanovo and PEAKS dropped by about 20%. Overall, Casanovo seems to be the most sensitive to the number of mutations, followed by PEAKS, GraphNovo, and PointNovo. We observed a similar trend to the results of the previous datasets that Casanovo had higher peptide accuracy but fell behind the other tools on the amino acid accuracy. We also calculated the *de novo* accuracies stratified by the proportion of mutated amino acids over the peptide lengths and found similar results (Supplemental Fig. S8).

We further investigated the *de novo* accuracy of mutated amino acids stratified by the positions. For this investigation, we considered the peptides with one mutated amino acid. Since the peptides have different lengths, we first looked at the peptides of length 15 (the most common length in the dataset). As shown in Figure 5*C*, the accuracy seemed to drop at the first and last three positions when compared to other middle positions of the peptides. Thus, we further calculated the accuracy of mutated amino acids located at the first three, the last three, and the middle three positions of all peptides in the dataset. The results in Figure 5*D* confirmed the accuracy drop at the first three and the last three positions. Further inspection of the spectra revealed that this was due to the missing fragment ions at the N-terminal and C-terminal, which led to the issue of adjacent amino acid swaps (*e.g.*, PEPTIDE and PEPTIED) or different amino acid combinations with the same total mass. Figure 5*D* also shows that GraphNovo was able to resolve this problem better than the other tools, as their authors have noted in Mao *et al*. (11).

It should be noted that Prosit-predicted spectra only contain major b/y-ions and they are different from experimental spectra. However, since the purpose of this analysis was to investigate the performance of *de novo* sequencing tools on mutated peptides, spectrum prediction was the only way to obtain the spectra of mutated peptides. If major b/y-ions are presented in the spectra, mutated amino acids should be identified. We did not introduce noise to the predicted spectra because it was not clear how the noise distribution should be simulated to be comparable to experimental spectra. We believe the difference between predicted and experimental spectra is a complicated problem, and, in fact, most studies on spectrum prediction often focus on the intensities of major b/y-ions, but not the noise. Thus, we decided to keep the mutation analysis simply on the predicted spectra.

### Deep Learning Models and Peptide Sequences

One of the concerns regarding deep learning–based *de novo* sequencing models is that they may memorize peptide sequences from their training data. In this section, we performed additional analyses to investigate this issue. We first checked the ABRF dataset and found that 82% of its PSMs could be found in the MassIVE-KB training data. We further compared the *de novo* accuracies on the PSMs found in MassIVE-KB and those not in MassIVE-KB. As shown in Supplemental Fig. S9, *A* and *B*, there was a substantial drop of accuracy on those PSMs not in MassIVE-KB. However, the drop was also observed on nondeep learning tools such as PEAKS and Novor. We further investigated those PSMs and found that their identification scores (by database search engine) were much lower than those PSMs found in MassIVE-KB. This indicates that the PSMs not in MassIVE-KB were low-quality spectra. Thus, this comparison was not a fair evaluation of the issue of memorizing peptide sequences because it involved the quality of the spectra.

To provide a fair evaluation for the issue of memorizing peptide sequences, we further generated three new datasets of three species, *E. coli*, yeast, and human. The datasets were generated under the same experimental conditions, instrument, and LC-MS/MS analysis. Thus, the only difference between them was the peptide sequences. We performed a database search on those three datasets and obtained 30-34K PSMs per dataset, with similar identification scores and peptide lengths distributions. About 96% of the human PSMs were found in MassIVE-KB, whereas only 0 to 2% of the *E. coli* and yeast PSMs were found in MassIVE-KB. We then compared the *de novo* accuracies on these three datasets. As shown in Supplemental Fig. S9, *C* and *D*, PEAKS, PointNovo, and Novor results show almost identical accuracies across three datasets. However, Casanovo and GraphNovo results show a drop of up to 3 to 4% of peptide accuracy on *E. coli* and yeast, as compared to human. While this drop might not seem to be a strong evidence, it still indicates a possibility of memorizing peptide sequences, especially when compared to the other three tools. It should be noted that, although PointNovo is also a deep learning–based tool, in this study, we did not apply its LSTM option, so it did not model the patterns of amino acids and peptide sequences like Casanovo and GraphNovo.

## Discussion

In this study, we proposed NovoBoard, a comprehensive framework to evaluate the performance of *de novo* peptide sequencing methods. The framework aims to establish a standard set of benchmark datasets and metrics that can thoroughly assess the strengths and weaknesses of *de novo* peptide sequencing methods and also their specific applications. Special focuses were put into addressing deep learning-based models to validate that they were truly able to generalize and discover new sequences, rather than overfitting and memorizing the training data. We also proposed a new method for FDR estimation and validation of *de novo* peptide-spectrum matches. Overall, the results in our study revealed important characteristics of different *de novo* peptide sequencing methods that will be valuable for future applications. The results also confirmed that such a comprehensive evaluation framework is crucial and needs more attention from the research community.

One limitation of our study is that the benchmark datasets mainly came from Orbitrap instruments and DDA experiments with HCD fragmentation. Due to limited time and resource constraints, we did not perform training and testing on the low-resolution and the nine-species datasets as in some previous studies (5, 8, 9, 11, 12). In this study, PointNovo, Casanovo, and GraphNovo models were all trained on the MassIVE-KB database, which we believe represents the most comprehensive training data to date and is suitable to cover a wide range of MS applications. Certainly it would be interesting to explore whether other types of MS data may have different effects on the performance of *de novo* sequencing tools and on the FDR estimation. The evaluation can be extended to data from other types of instruments or fragmentation techniques, but more importantly, the deep learning models will need to be re-trained. We did a quick test on an ETD dataset (27) and found that PointNovo, Casanovo, and GraphNovo failed to produce any meaningful results, while PEAKS and Novor, still performed reasonably well. Thus, this topic is a good direction for future research studies. In addition, DIA data also represents a challenging problem for *de novo* peptide sequencing. Currently, *de novo* peptide sequencing methods for DIA data are not very popular and their evaluation may be more complicated than for DDA data.

In this study we did not perform a detailed analysis on the computing time and resources of the *de novo* sequencing tools. We believe that it is more related to the engineering efforts and the available hardwares. Nevertheless, we did a quick test on the ABRF dataset and reported here a rough estimation of the tools’ running time: PEAKS 100 spectra/second (on CPU i9-13900K), PointNovo 110 spectra/second, Casanovo 150 spectra/second, and GraphNovo 273 spectra/second (the later three were run on GPU, NVIDIA GeForce RTX 4090). It is worth noting that a speed of ≥ 250 spectra/second should be able to keep up with the data acquisition speed of most MS instruments and enable real-time *de novo* sequencing directly on the instruments.

## Data Availability

The raw data of the previously published datasets can be obtained from the ProteomeXchange Consortium *via* the PRIDE (21) partner repository with the dataset identifiers PXD010154, PXD013658, and PXD021013. The new MS data, database search, and *de novo* sequencing results of our study have been deposited to the ProteomeXchange Consortium *via* the PRIDE partner repository with the dataset identifier PXD055277. This also includes the decoy spectra, the dataset of mutated peptides, and all MGF files that we generated in this study. The Python scripts for calculating the fragment ion, amino acid, peptide accuracy metrics, and for generating decoy spectra, FDR estimation, and validation are available on GitHub *via* the following link https://github.com/nh2tran/NovoBoard.

## Supplemental data

This article contains supplemental data.

## Conflicts of interests

N. H. T., R. Q., Z. M., S. P., Q. Z., W. L., L. X., and B. S. are employees of Bioinformatics Solutions Inc.
