# Supplementary material for: NovoBoard: A Comprehensive Framework for Evaluating the False Discovery Rate and Accuracy of De Novo Peptide Sequencing
Source: Mol Cell Proteomics. 2024 Sep 24;23(11):100849. doi: 10.1016/j.mcpro.2024.100849 (PMC11532909; doi:10.1016/j.mcpro.2024.100849)
Supplement: Supplemental data [file mmc1.pdf]

NovoBoard: a comprehensive framework for evaluating the false discovery rate and accuracy of de novo peptide sequencing

Ngoc Hieu Tran[1,*], Rui Qiao[1,*], Zeping Mao[1,2,*], Shengying Pan[1,*], Qing Zhang[1], Wenting Li[1], Lei Xin[1,**], Ming Li[2,**], Baozhen Shan[1,**]

[1] Bioinformatics Solutions Inc., Waterloo, Ontario, Canada.

[2] David R. Cheriton School of Computer Science, University of Waterloo, Ontario, Canada.

* These authors contributed equally to this work.

** Corresponding authors. Emails: lxin@bioinfor.com, bshan@bioinfor.com, mli@uwaterloo.ca

This Supplemental Data includes Supplementary Figures S1-S9.

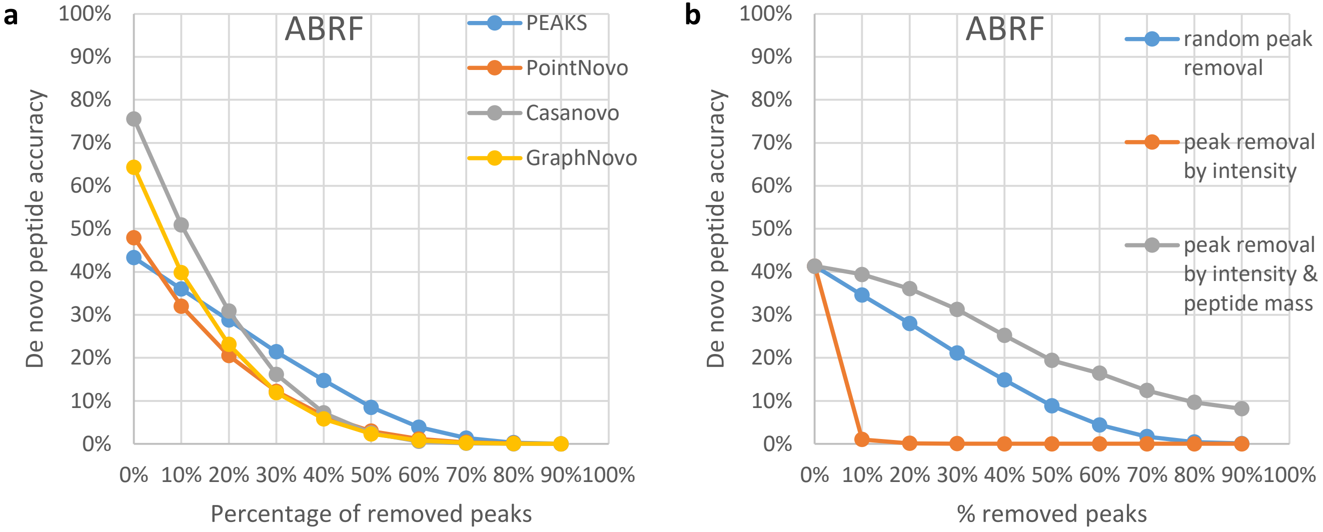

Supplementary Figure S1. a, De novo peptide accuracy with respect to the percentage of randomly removed peaks in the decoy spectra generated from the ABRF dataset. **b,** De novo peptide accuracy of PEAKS with respect to different strategies of peak removal.

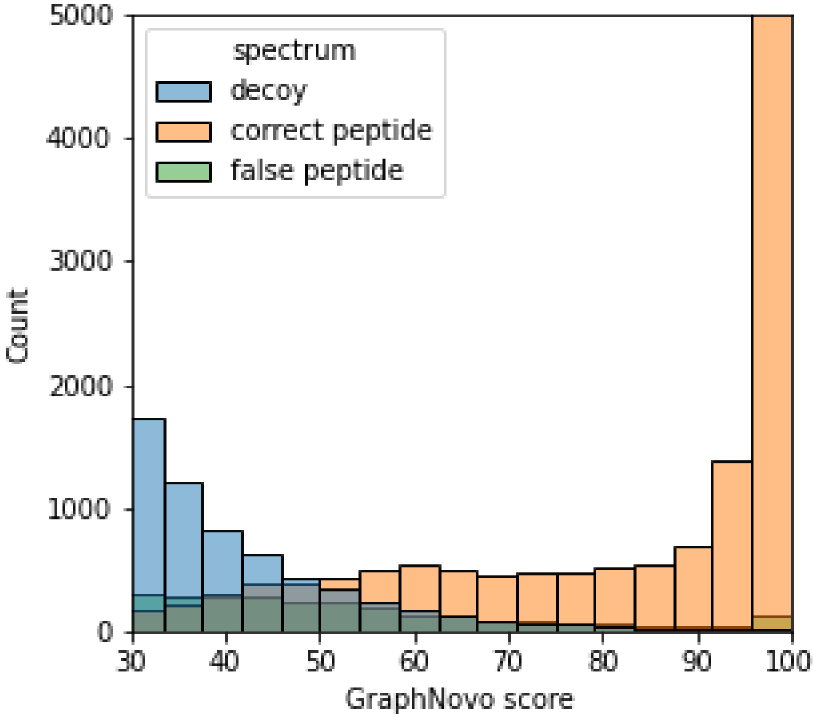

Supplementary Figure S2. GraphNovo score distributions of decoy PSMs and target PSMs with correct and false de novo peptides from the ABRF dataset. The decoy spectra were generated by randomly removing 60% of the target peaks and replacing them with noise peaks.

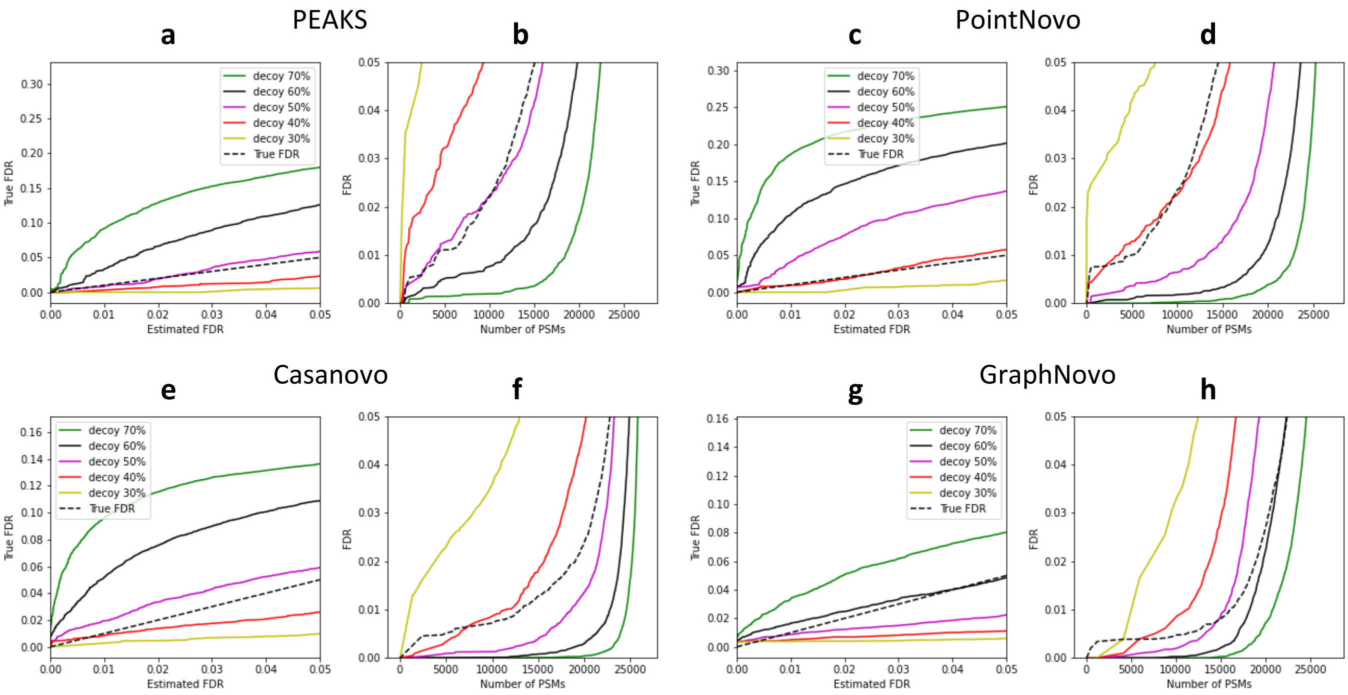

Supplementary Figure S3. FDR estimation and validation using the decoy spectra generated from the ABRF dataset. In this example, the decoy spectra were generated by random peak removal. Different colors indicate different percentages of removed peaks, for instance, decoy 70% (green) means that we removed 70% of the original peaks in a target spectrum and replaced them with noise peaks to generate the corresponding decoy spectrum. For each de novo sequencing tool, the estimated FDR, the true FDR, and the number of de novo PSMs were calculated with respect to different de novo score cutoffs.

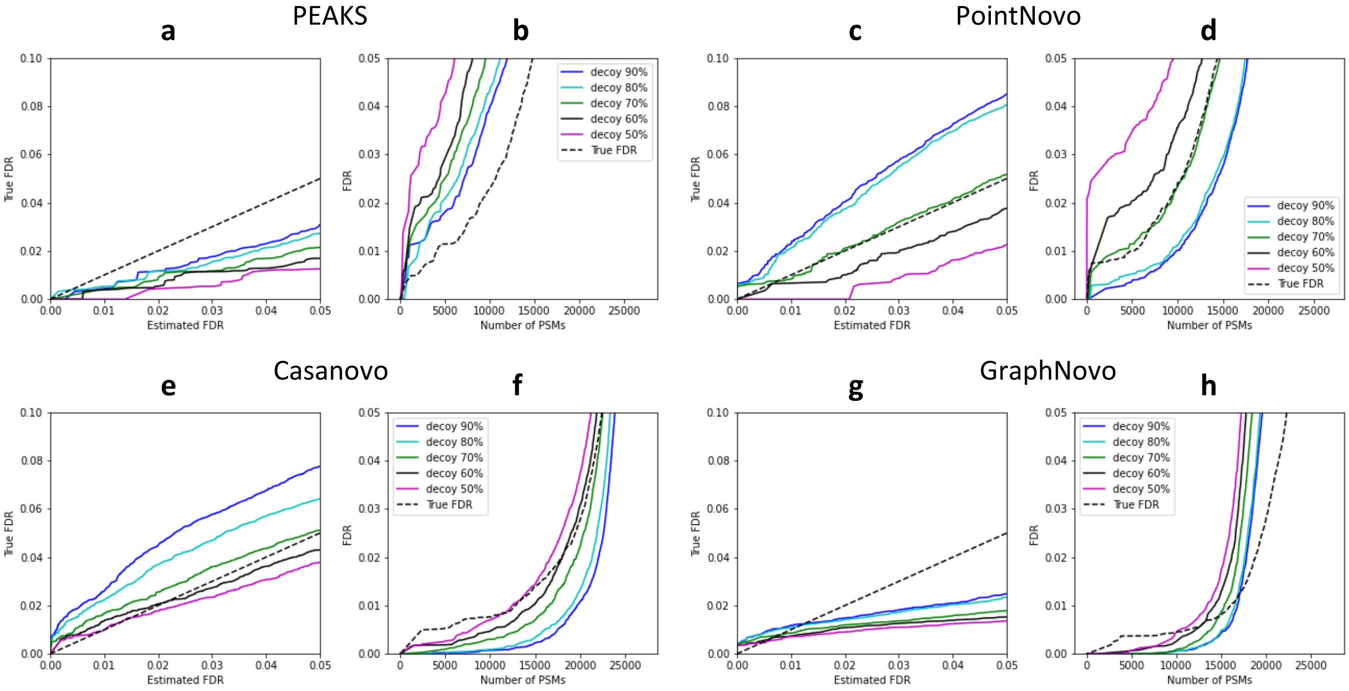

Supplementary Figure S4. FDR estimation and validation using the decoy spectra generated from the ABRF dataset. In this example, the decoy spectra were generated by removing peaks by their intensities and the peptide mass. Different colors indicate different percentages of removed peaks, for instance, decoy 70% (green) means that we removed 70% of the original peaks in a target spectrum and replaced them with noise peaks to generate the corresponding decoy spectrum. For each de novo sequencing tool, the estimated FDR, the true FDR, and the number of de novo PSMs were calculated with respect to different de novo score cutoffs.

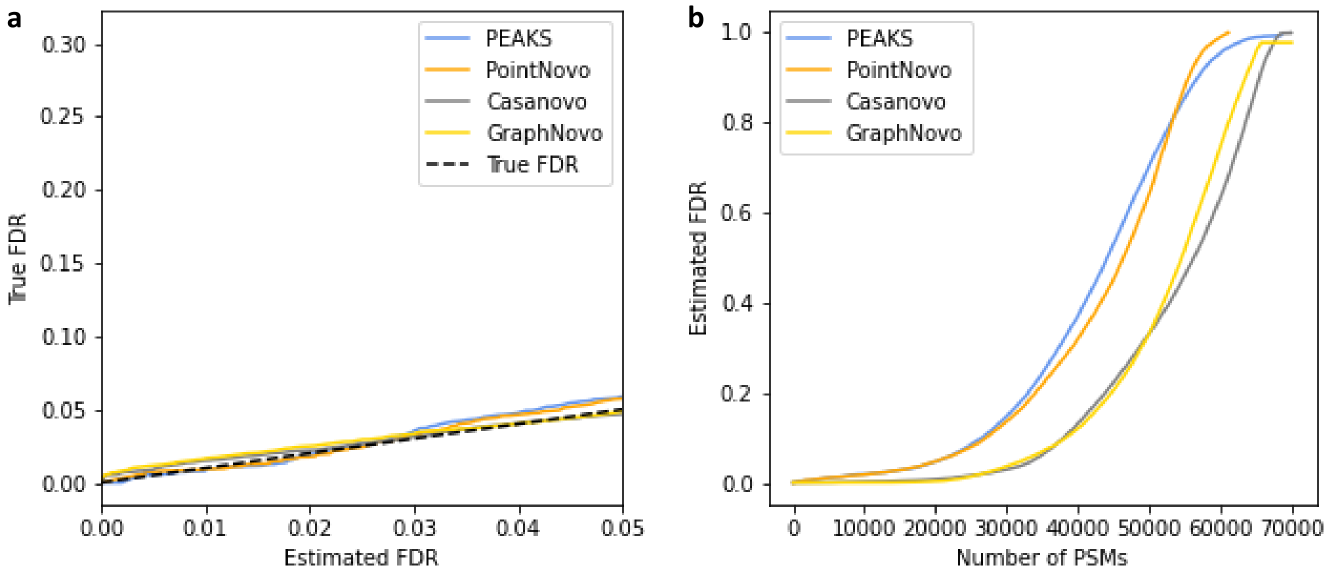

Supplementary Figure S5. a, Estimated FDR versus the true FDR of four de novo sequencing tools on the ABRF dataset and its decoy spectra. The decoy spectra were generated by randomly removing target peaks and replacing them by noise peaks from the same peak distribution. The percentages of removed peaks were selected specifically for each tool so that its estimated FDR was close to the true FDR, i.e. PEAKS 50%, PointNovo 40%, Casanovo 45%, and GraphNovo 60%. **b**, The number of de novo PSMs reported by the four tools with respect to the estimated FDR on the entire ABRF dataset (i.e. including the spectra without ground-truth peptides).

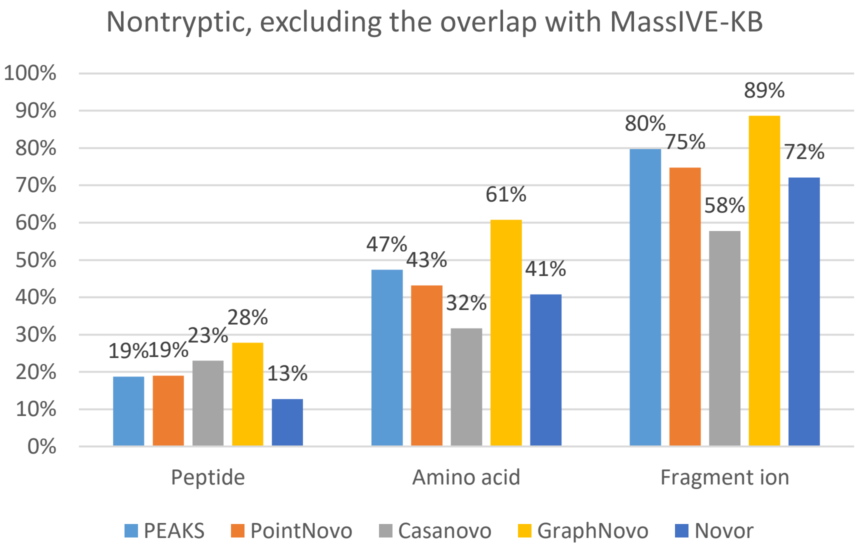

Supplementary Figure S6. Evaluation of the de novo results on the nontryptic dataset, excluding the overlap with MassIVE-KB.

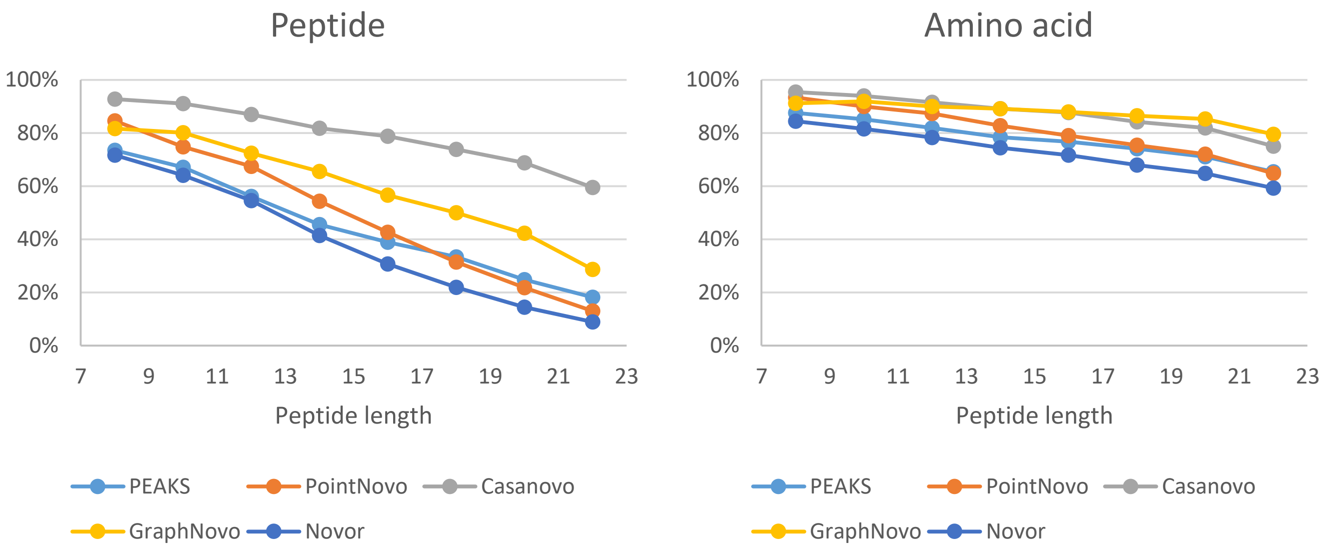

Supplementary Figure S7. Evaluation of the de novo results on the ABRF dataset stratified by peptide lengths.

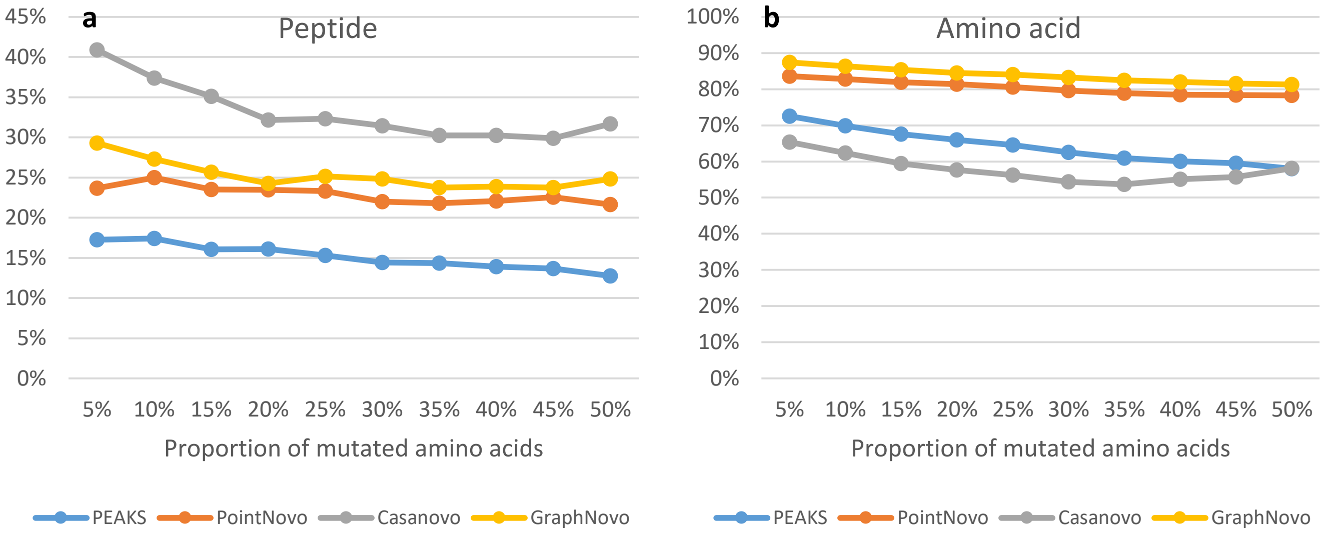

Supplementary Figure S8. Evaluation of the de novo results on the simulated dataset of mutated peptides. **a-b,** Peptide and amino acid accuracies with respect to the proportion of mutated amino acids.

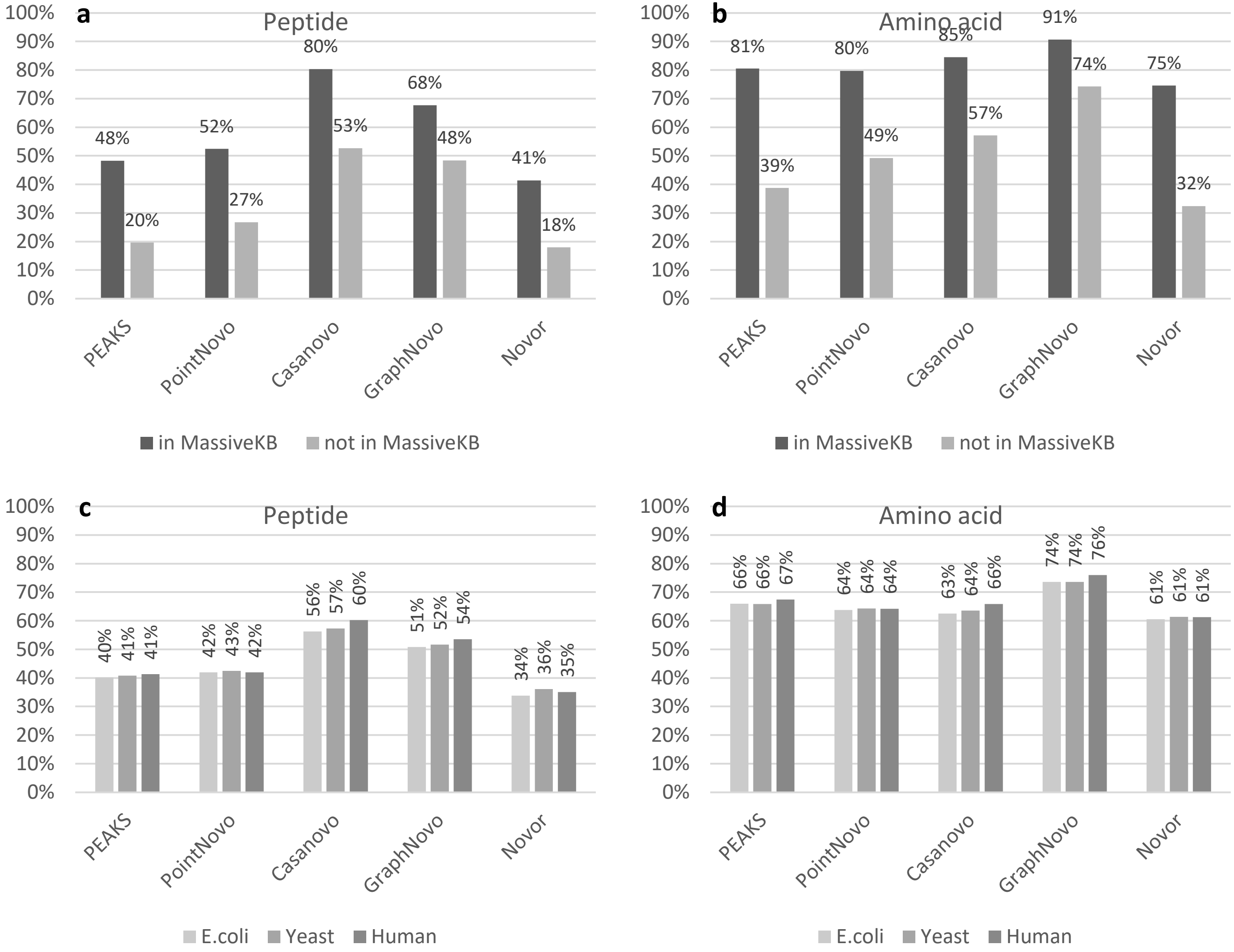

Supplementary Figure S9. Analysis of the issue of "memorizing peptide sequences" of the de novo sequencing tools. **a-b,** Results on the ABRF dataset. **c-d,** Results on the three species datasets generated in our study.
